# Dynamic cluster scheduling for cluster-tree WSNs

**DOI:** 10.1186/2193-1801-3-493

**Published:** 2014-08-31

**Authors:** Ricardo Severino, Nuno Pereira, Eduardo Tovar

**Affiliations:** CISTER Research Centre, ISEP/IPP, Rua Antonio Bernardino de Almeida 431, 4200-072 Porto, Portugal

**Keywords:** Cluster-tree networks, Message scheduling, Quality-of-service in WSN

## Abstract

While Cluster-Tree network topologies look promising for WSN applications with timeliness and energy-efficiency requirements, we are yet to witness its adoption in commercial and academic solutions. One of the arguments that hinder the use of these topologies concerns the lack of flexibility in adapting to changes in the network, such as in traffic flows.

This paper presents a solution to enable these networks with the ability to self-adapt their clusters’ duty-cycle and scheduling, to provide increased quality of service to multiple traffic flows. Importantly, our approach enables a network to change its cluster scheduling without requiring long inaccessibility times or the re-association of the nodes. We show how to apply our methodology to the case of IEEE 802.15.4/ZigBee cluster-tree WSNs without significant changes to the protocol. Finally, we analyze and demonstrate the validity of our methodology through a comprehensive simulation and experimental validation using commercially available technology on a Structural Health Monitoring application scenario.

## Introduction

The increasing tendency for the integration of computations with physical processes at large scale has been pushing research on new paradigms for networked embedded systems design (Stankovic et al. 2005). In this line, Wireless Sensor Networks (WSNs) have naturally emerged as enabling infrastructures for these cyber-physical applications due to their potential to closely interact with external stimulus. Applications such as homeland security, health care, building or factory automation are just a few elucidative examples of how these emerging technologies will impact our daily life and society at large.

Given the large number of these WSN applications, each with an individual set of requirements (Raman and Chebrolu 2008), it is important that some of these WSN resources (e.g. bandwidth and buffer size), are predicted in advance, in order to support the prospective applications with a pre-defined Quality-of-Service (QoS). To achieve this, it is mandatory to rely on structured logical topologies such as cluster-trees (e.g. (Abdelzaher et al. 2004, Gibson et al. 2007, Prabh and Abdelzaher 2007)), which provide deterministic behaviour instead of flat mesh-like topologies, where QoS guarantees are difficult to provide, if not impossible.

Nevertheless, although these network topologies look promising for the above mentioned WSN applications, there is a lack of flexibility in adapting to changes in the traffic or bandwidth requirements at run-time, making them not capable of allocating more bandwidth to a set of nodes sensing a particular phenomena, or reducing the latency of a data stream. In fact, although there is already some literature on how to compute these network resources (Jurcik et al. 2010, Hanzalek and Jurcik 2010), it is not clear how they could be re-allocated without greatly interfering with the network functionality, and specially without imposing high inaccessibility times.

This paper presents a solution to this problem, enabling networks to change at run-time a given initial schedule, based on a time-division strategy, to provide increased quality of service to multiple traffic flows. Computing this would normally result in a complex integer programming problem which would be infeasible to be computed by WSN nodes which typically have scarce computing power. Our re-scheduling algorithm relies on a heuristic that can be easily computed in these platforms. We show how to apply our methodology to the particular case of IEEE 802.15.4/ZigBee, good candidates to enable this kind of networks. Finally, we analyze and demonstrate the validity of our methodology through a comprehensive simulation study and experimental validation using WSN platforms in a real-world Structural Health Monitoring scenario. Our proposal can reduce the end-to-end latency by 93% and the overall data stream transmit period by 49%, although higher values can be achieved under different network settings.

## Related work

In general, synchronized Cluster-Tree topologies tend to suffer from four technical issues that usually prevent their use: (1) how to schedule the transmissions of different neighbouring clusters avoiding interference; (2) how to predict the performance limits to correctly allocate resources; (3) how to change the resource allocation of the Cluster-tree (CT) on-the-fly; and (4) the lack of available and functional implementations over standard WSN technologies, such as the IEEE 802.15.4/ZigBee set of protocols.

There is already an interesting body of work concerning the scheduling of general tree-based WSNs. Most of the work addresses the case of minimizing the length of TDMA-based schedules for improved convergecast (Choi et al. 2009, Lai et al. 2008). In (Gandham et al. 2008), a distributed algorithm is proposed in contrast with previous more centralized solutions. Recently, in (Incel et al. 2012) a scheduling strategy is combined with transmission power control to minimize collision between nodes, and a strategy to schedule transmissions in different frequencies is also proposed.

Although these strategies might work for a pure TDMA-based tree, cluster-based trees impose a different approach since each slot of the TDMA cycle is usually not allocated to one single node, but to a cluster with many nodes, and often nodes which are contending for medium access, thus rendering most delay bound results not significant. This greatly reduces the number of application scenarios for such proposals, considering current standardization trends.

This is especially true for the particular case of the IEEE 802.15.4/ZigBee set of protocols, in which although the Cluster-Tree network topology is supported, no clear description on how to implement it is given, namely in what concerns the beacon collision problem. In (Std. IEEE 802.15.4 2006), the Task Group 15.4b proposed some basic approaches to solve this: the beacon-only period approach and the time division approach, only to be removed in the 2006 revision.

In this line, a few proposals were made targeting the scheduling of ZigBee cluster-tree networks. The work in (Pan and Tseng 2008) introduced the Minimum Delay Beacon Scheduling problem, however this proposal only addressed the latency problem and not the bandwidth problem, since it assumed the use of GTS slots for convergecast. The work in (Jurcik et al. 2010), addresses the problem of predicting resource needs by modelling the performance limits of a ZigBee CT network using GTS flows. In another proposal (Hanzalek and Jurcik 2010), the authors extend the previous work by computing the optimal schedule for several GTS data flows. Recently, (Di Francesco et al. 2012) followed a similar approach to (Pan and Tseng 2008), proposing two heuristics to reduce the complexity of the otherwise NP-complete problem. Although the usage of GTS guarantees real-time performance within the IEEE802.15.4/ZigBee standards, the number of available GTS slots is quite limited as well as their bandwidth. In this line, in (Huang et al. 2012) the authors try to overcome this by borrowing bandwidth from neighbouring nodes.

All of the above proposals work by computing a static schedule, based on periodic traffic assumptions, which will remain active throughout the network lifetime. Moreover, they follow a purely theoretical approach, lacking a clear description on how to implement such mechanisms on ZigBee. In fact, in some cases it is arguable if it is even possible.

For instance, the work in (Huang et al. 2012) proposes the concept of adoptive-parents, something which is clearly not compliant with the ZigBee protocol. Similarly, two other proposals (Yeh et al. 2008) and (Dan et al. 2010), try to improve routing efficiency and decrease latency by proposing important changes to the basis of these standards. The first by proposing a change to the superframe structure to encompass two active periods per Cluster-Head, the second, by proposing a completely new tree-routing protocol for ZigBee. In (Kim et al. 2007) the authors propose yet another non-compliant way of reducing the schedule latency by passing frames to neighbouring clusters, changing a cluster-tree topology into a mesh by supporting multiple paths. Moreover, allowing inter-cluster messaging leads to interference and eventually beacon collision problems, since nodes do not know the neighbouring cluster’s active periods. In (Toscano and Lo Bello 2009) the authors use different radio channels to avoid tackling the problem.

It is clear that standard communication technologies able to support tree-based topologies, could benefit from full-compliant scheduling mechanisms. To make this a reality, proposals should as much as possible, present clear implementation details, showing how to enable their usage within current communication standards. In these authors’ opinion, in addition to simulation, carrying out experimental validations of such mechanisms over real-world platforms is mandatory when addressing these protocols.

In this line, in (Koubaa et al. 2007), the Time Division Cluster Schedule (TDCS) algorithm was proposed and implemented in the Open-ZB stack (Cunha et al. 2007) enabling for the first time the use of this topology over IEEE 802.15.4/ZigBee based networks, guaranteeing no beacon collisions. This technique used a time-division approach and worked by assigning a different time offset to each cluster. Fully implemented over commercial WSN platforms, available to the TinyOS community (TinyOS 2014), and with a set of network planning tools available to the general WSN community via Open-ZB (Open-ZB 2014), we believe this work to be a proven reference concerning beacon scheduling for CT ZigBee networks. Other proposals followed a similar approach such as (Burda and Wietfeld 2007) for mesh networks, or (Muthukumaran et al. 2010).

Although some literature on solving the first two aforementioned problems in this section is already available, none of the proposals so far, in the general case of synchronized Cluster-Trees, addresses the third one, at least in a satisfactory way, and guaranteeing standard compliance. This greatly limits the flexibility of the network which must keep the same cluster schedule and bandwidth reservation, independently of the flow of data in the network and of its particular requirements, which depending on the application may certainly change. In this paper, we propose a set of techniques in which the base schedule is temporary changed to encompass transient networking necessities such as end-to-end delay and bandwidth allocation. This work, already presented in (Severino et al. 2013a), is now extended with new experimental results obtained over a real-world structural health monitoring application and significant more detail is given to the proposal and its implementation over the IEEE 802.15.4/ZigBee standards.

## System model

Consider a synchronized cluster-tree WSNs featuring a tree-based logical topology where nodes are organized in different groups, called clusters. Each node is connected to a maximum of one node at the lower depth, called parent node, and can be connected to multiple nodes at the upper depth, called child nodes (by convention, trees grow down). Each node only interacts with its pre-defined parent and child nodes.

**Figure 1:**
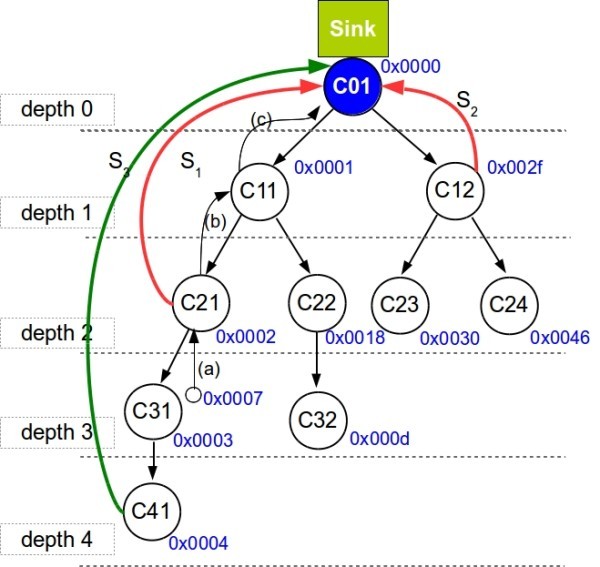
**System model.**

A cluster-tree topology contains two main types of nodes: routers and end-nodes (refer to Figure 1). The nodes that can associate to previously associated nodes and can participate in multi-hop routing are referred to as routers. The leaf nodes that do not allow association of other nodes and do not participate in routing are referred to as end-nodes. The router that has no parent is called root and it might hold special functions such as identification, formation and control of the entire network. Note that the root is at depth zero. Both routers and end-nodes can have sensing capabilities, therefore they are generally referred to as sensor nodes. Each router forms its cluster and is referred to as cluster-head of this cluster (e.g. router *C*_11_ is the cluster-head of cluster 11). Each cluster-head is also responsible for synchronization in its cluster and periodically sends synchronization frames. All child nodes (i.e. end-nodes and routers) of a cluster-head are associated to its cluster, and the cluster-head handles all their data transmissions. It results that each router (except the root) belongs to two clusters, once as a child and once as a parent (i.e., a cluster-head). A schedule of the clusters to minimize or eliminate inter-cluster interference, following a time-division strategy is assumed to be already in place.

In general, the radio channel is a shared communication medium where more than one node can transmit at the same time. In cluster-tree WSNs, messages are forwarded from cluster to cluster until reaching the sink. The time window of each cluster is periodically divided into an active portion (AP), during which the cluster-head enables data transmissions inside its cluster, and a subsequent inactive portion, during which all cluster nodes may enter low-power mode to save energy resources. Note that each router must be awake during its active portion and the active portion of its parent router. To avoid collisions between clusters, it is mandatory to schedule the clusters’ active portions in an ordered sequence, that we call TDCS so that no inter-cluster collision occurs. In case of single collision domain, the TDCS must be non-overlapping, i.e. only one cluster can be active at any time. Hence, the duration of the TDCS’s cycle is given by the number of clusters and the length of their active portions. On the contrary, in a network with multiple collision domains, the clusters from different non-overlapping collision domains may be active at the same time. However, finding a TDCS that avoids clusters’ collisions in a large-scale WSN with multiple collision domains is a quite complex problem, hence in this paper, for simplification, we always assume a single collision domain. For more information concerning TDCS, please refer to (Koubaa et al. 2007).

Several data transmissions in an upstream direction (e.g. streams *S*_1_,*S*_2_,*S*_3_ in Figure 1) can be present in the network simultaneously. Each stream is noted as a tuple *S*_*k*_=<*R*_*k*_,*P*_*k*_,*T*_*k*_,*D*_*k*_>, where, *R*_*k*_ represents the ordered set of clusters which the stream *k* must cross to reach the sink, *P*_*k*_ represents the priority for that stream (an integer from 0 to 5), *T*_*k*_ represents the number of TDCS cycles for which stream *k* will remain active and *D*_*k*_ the depth of the stream’s source. This stream notation will be used in the next section to support the computation of a better TDCS schedule to apply to the network.

## Dynamic cluster scheduling

With TDCS (Koubaa et al. 2007), it is possible to find the best schedule for the routers active periods in order to avoid interference, and to support most of the network bandwidth requirements. However, the schedule is done at network setup time and assumes a static network that will remain unchanged. The choice of the TDCS schedule has a strong impact on the end-to-end delays. In fact, it is easy to observe that in a single collision domain, where there are no overlapping clusters, a TDCS schedule optimized for downstream communication will result in the worst-case for upstream communication, and consequently in higher end-to-end delays. Moreover, routers are assigned with a fixed bandwidth they might not always need, while other clusters might be lacking. We aim at reacting to different data flow changes on-the-fly, while simultaneously minimizing the network inaccessibility time. Our proposal achieves this via two techniques: (1) re-ordering the clusters’ active periods to favour one set of streams, reducing the end-to-end delays, which we call DCR (Dynamic Cluster Re-ordering); and (2) tuning the size of the clusters’ duration, increasing the bandwidth of the clusters serving a specific stream, an eventually decreasing others’ bandwidth, which we named DBR (Dynamic Bandwidth Re-allocation). The first technique consists of a rescheduling of the clusters order in the TDCS cycle, aiming at minimizing end-to-end delays, while the second technique consists on rearranging the bandwidth allocation for the clusters involved in a stream, to increase its bandwidths and decrease the overall time for a data stream transmission. Both techniques can be used together, or separately. Importantly, the mechanism presents a complexity of *O*(*N*), where *N* represents the number of Cluster-Heads in the network, making it suitable to be run over WSN platforms with scarce processing power. This low complexity also avoids a much larger energy depletion of the central node in charge of running DCS.

### Dynamic cluster re-ordering

**Figure 2:**
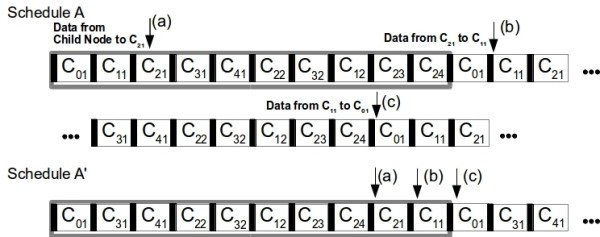
**Cluster schedule.**

Consider the cluster-tree presented in Figure 1, with 10 clusters and a TDCS schedule as presented in Figure 2 Schedule A, where all CHs have the same allocated bandwidth. Notice that this schedule is set to minimize downstream traffic latency (parents’ appear earlier in the schedule than the child nodes), which is common in applications that require tight actuation. In this way, to act on Cluster *C*_21_ for instance, one could do it in only one TDCS cycle, since those Cluster’s are active immediately one after the other. However, to receive data from *C*_21_, assuming that all data could be transmitted from one CH to the next in one TDCS cycle, it would take two cycles, one from *C*_21_ to *C*_11_, and another from *C*_11_ to *C*_01_. This is depicted in Figure 2, where (a) represents the data coming from the sensing node and being received by *C*_21_, (b) represents the transmission from *C*_21_ to *C*_11_, and finally, (c) from *C*_11_ to *C*_01_. This delay will increase as the network size and the clusters’ duration increases and as the depth of the source increases. In this scenario, the best schedule to minimize upstream latency, considering a stream from Cluster *C*_21_ to the Sink (*S*_1_ in Figure 1), should be as depicted in Figure 2 Schedule A’, where the next cluster to receive the packet appears next, reducing the amount of time a packet needs to be left in the queue and consequently the application end-to-end delays. Thus, networks should carry out an on-line re-scheduling of the clusters to favour a known set of upstream data streams, minimizing the latency.

This kind of rescheduling involves a re-ordering of the Clusters according to the streams the network must serve. This can easily grow into a complex problem if one wishes to achieve an optimum solution, due to the clusters’ precedence in the tree, usually solved in the literature using integer programming as seen in the proposal in Section “Related work”. However, in order to react to the network specific needs in a reasonable amount of time, one needs to guarantee that the algorithm to compute this new schedule is light and fast enough to be run in WSN platforms with scarce processing power. In this line, integer programming models might not be the best choice for this case, where we just need a better and not necessarily the optimum solution. Our approach to the problem is explained next.

As already presented in Section “System model”, each stream is noted as a tuple *S*_*k*_=<*R*_*k*_,*P*_*k*_,*T*_*k*_,*D*_*k*_>. Given the set of streams *S*, and the set *N* which contains all the cluster-heads in the tree, we must compute *A* which denotes the set of cluster-heads that need rescheduling, where *A*=*N*∩*S*. Then, *C*_*r*_, which denotes the priority of the *r*^*th*^ cluster-head in *N*, can be computed through the following algorithm:


In other words, being *M*_*r*_ the set of streams from *m* to *N*_*m*_ which contain CH *r* in *R*, and *P*_*m*_ the stream’s priority, we can compute *C*_*r*_ as:
1

Function *h*(*A*_*r*_) computes the height of Cluster-Head *A*_*r*_ in the tree, according to the position each Cluster holds in array *R*_*k*_ being the first CH in the array position 0. Thus,
2

This value will add to the already computed cluster’s priority to enable precedence in the schedule. The resulting schedule will be achieved by ordering the set of all cluster-heads *N* according to the computed *C*_*r*_ for each cluster-head *A*_*r*_, starting from the lower priorities to the highest. As a result, the highest priority will always be assigned to the sink, since all the streams are directed to that cluster-head. Cluster-heads that are not part of the set *A* keep their schedule not to interfere with the initial schedule of those, and are placed after the sink. As an example, if we consider the network presented in Figure 1 and assume the following set of streams: *S*_1_=<{*C*_21_,*C*_11_,*C*_01_},3,3,2> and *S*_2_=<{*C*_12_,*C*_01_},1,4,1>, *A* would be *A*={*C*_01_,*C*_11_,*C*_12_,*C*_21_}.

The first stream, originates at cluster *C*_21_ and has priority 3, while the second, originates at cluster *C*_12_ and has priority 1. If no reschedule was done, and assuming ideal communication without errors and delays imposed by the MAC layers, we would expect that one packet of *S*_1_ would take approximately 18 times the duration of one active portion of a CH to reach the sink (Figure 2 Schedule A), and from *S*_2_ three active portions. If we use the presented algorithm it will result in the following: *C*_01_=*P*_1_+*P*_2_+*h*(*A*_01_)=3+1+2=6; *C*_11_=*P*_1_+*h*(*A*_11_)=3+1=4; *C*_12_=*P*_2_+*h*(*A*_12_)=1+1=2; *C*_21_=*P*_1_+*h*(*A*_21_)=3+0=3; Ordering from the lowest to the highest priority, the CHs in *A* should be ordered as *C*_12_,*C*_21_,*C*_11_ and finally *C*_01_.

**Figure 3:**
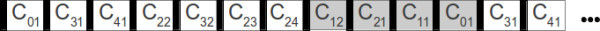
**Re-ordered DCR schedule.**

Considering the remaining nodes, which maintain their initial order in the schedule and lowest priority, the final schedule, would be as described in Figure 3.

It would now be possible a full data transaction from the origin cluster to the sink in one TDCS cycle, reducing the delay of each packet, greatly benefiting applications which demand low latencies. If we wanted to decrease the latency for *S*_2_ we could increase the priority of the stream to the same of *S*_1_ or higher. This would result in *C*_12_=*P*_1_+*h*(*A*_12_)=3+1=4, and now, *C*_12_ would have a higher priority than *C*_21_ thus appearing later in the schedule, decreasing the latency.

Comparing this schedule with the original in Figure 2 Schedule A, we observe that the other CHs also changed place in the schedule. Changing the position of all nodes must be done because there is no free room that will let us only change the streaming CHs’ position and accommodate their initial positions unoccupied. However, this does not mean that all of the CHs changed the offset to their parents. For instance, in this particular case *C*_41_ does not change the offset. This is obvious, since the distance between *C*_41_ and its parent *C*_31_ did not change. As a rule of thumb, a new offset will have to be computed for every children one depth bellow a re-scheduled CH. For their grand-children, this does not happen since the distance remains the same as in the original schedule. This principle will be used later in STEP 4 (Section “The DCS communication protocol”), to compute the network’s inaccessibility time.

Although this approach solves the latency problem, it does not reduce the overall time it will take for a stream to be transmitted since there is no change to the available bandwidth per cycle. Hence our second proposal, DBR, which will increase the bandwidth for the clusters involved in the stream.

### Dynamic bandwidth re-allocation

**Figure 4:**
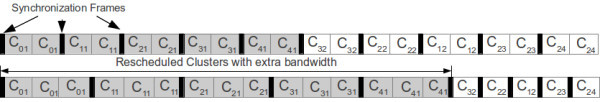
**DBR Schedule.**

For this technique, bandwidth must be re-allocated by increasing the bandwidth for the clusters involved in the stream. The first step is to look for free space in the schedule that has not been reserved by a cluster’s active portion. If there is such free space, we can distribute in an equal fashion the available space by the Clusters involved in the stream. For the particular case of Figure 2 Schedule A there is no space available. This means we must try to reduce the amount of bandwidth the clusters not related to the stream are using. Here, it is important to previously define the minimum bandwidth a Cluster can support. This is implementation specific in many cases, since it is highly dependent on the limitations of the hardware platforms. If the SO (Superframe Order - refer to Section "Instantiating DCS in IEEE 802.15.4/ZigBee") is reduced beyond a threshold, there can be timing issues. This has been reported previously and is discussed in (Cunha et al. 2008) concerning the TelosB platforms. The minimum bandwidth that will be available to the other clusters after the use of this technique is thus set at network setup time. If we consider stream *S*_3_ (Figure 1) originating a *C*_41_, in which the routers involved are *R*_41_={*C*_41_,*C*_31_,*C*_21_,*C*_11_,*C*_01_}, the one we wish to increase the bandwidth of every cluster, and a network which is capable of handling a reduction of the available bandwidth by half, this technique will cut all the remaining 5 CH’s duration, and redistribute this duration by the other CH’s in *R*_41_. This results in an increased bandwidth for that stream (Figure 4), thus reducing the transmission time. The size of the TDCS cycle is kept nonetheless, since the bandwidth was simply redistributed.

As depicted in Figure 4, all the relative offsets have changed. Nevertheless, a great plus of this technique is that the network inaccessibility time is minimum if compared to the previous technique, since in only one TDCS cycle, it is possible to reschedule all the network with the new offsets, if the original schedule was setup to facilitate downstream communications. This technique is, however, greatly dependent of the protocol in use since, some protocols only allow discrete steps in the duration of the CH’s active portion, like the IEEE802.15.4/ZigBee set of protocols. Refer to Section 5 concerning IEEE802.15.4/ZigBee protocols for more detail.

### The DCS communication protocol

**Figure 5:**
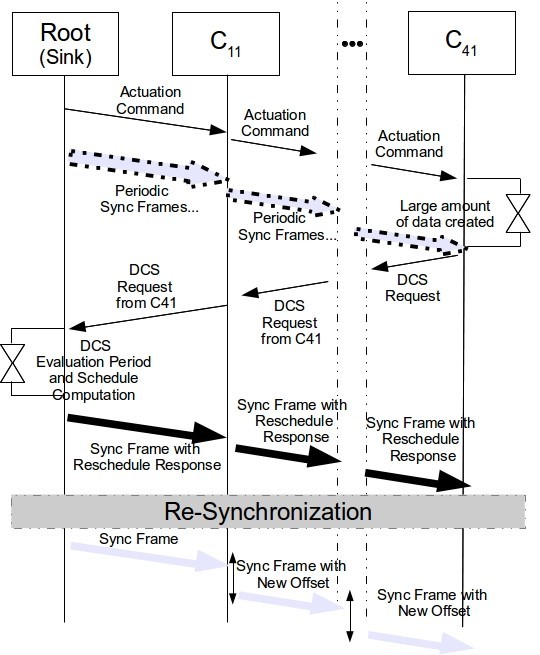
**DCS Communication diagram.**

Our proposed on-line re-scheduling technique comprises six steps, which can easily be adapted to different network protocols. The protocol is depicted in Figure 5 in a time diagram and is described next.

STEP 1 - At network setup time, all Cluster-Heads are assigned with a TDCS time offset in relation to their parents according to the approach proposed in (Koubaa et al. 2007). Different priorities are also assigned to different sensing actions by the nodes. Synchronization frames are sent periodically and several actuation actions on the leaf nodes can be carried out.

**Figure 6:**
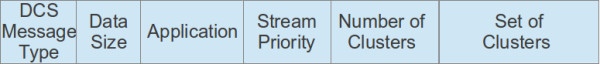
**DCS Request message format.**

STEP 2 - DCS Request; If a leaf node wishes to transmit a stream of data to the Sink, its Cluster-Head must be informed. The CH will decide, according to the application which originates the request, if the most adequate strategy is a rescheduling to minimize end-to-end delays, a reorganization of the bandwidth, or both. The option of which technique to use must be defined at network setup time, since different applications impose different requirements (reduced latency or transmission time). This request is then forwarded to the parent until it reaches the Root. On the way, each CH will add its own address to the message, to inform the Root of the clusters involved in the stream. This way, we avoid using heavy lookup tables that would have to be loaded into the Root at network setup time describing all parent child relationships. The DCS Request is shown in Figure 6. The first field transports the DCS Request message code identifier. Next, the estimated amount of data to be transmitted in the stream, and the application which is requesting the DCS.

The next fields identify the stream priority, for computing the new schedule, number of clusters which belong to the set, and their identification. These two last fields are updated as the DCS Request is transmitted upstream. Upon reception, the Root will wait for a finite period of time for more requests. It will then evaluate the Stream Requests and compute a new TDCS schedule.

STEP 3 - Evaluation and Rescheduling; The evaluation process consists in checking whether or not it is worth rescheduling the network, considering the amount of data to be transmitted and the inaccessibility time resulting from the reschedule. Although different techniques could be used to compute this, we are interested in speed and low complexity, due to the scarce processing power of common WSN platforms. The objective is to roughly compute the benefit from scheduling, and to do it fast enough not to delay the process too much. To compute this, we start by defining a base unit to simplify the computation. The base unit represents the duration of the active portion of the CH where a stream originates. Hence, if we say that a stream has size *n*=1, this represents a stream which duration is equal to the duration of its CH active portion. All the others CH durations can be represented as multiples of this base unit, because streams move upstream, thus the Bandwidth of the parent CHs, must be equal or higher than their child’s. This is imposed by the TDCS algorithm 2007. We also introduce the concept of *μcycle* and *macrocycle*. Here, the *μcycle* represents the amount of *n* units it takes for a stream of size *n*=1 to reach its destination and *macrocycle*, represents the size of the network TDCS schedule in multiples of *n*. The amount of time to transmit an amount of data represented in multiples of *n* can be computed using the following expression, where *T*_*i*_ represents the overhead of the rescheduling which we show how to compute in Step 5.
3

For the particular case of the network depicted in Figure 1, with schedule A, and considering a stream originating at *C*_41_ (*S*_3_), we can compute it’s *μcycle* as the number of base units between the different CHs in the path. The result is shown in Table 1.

**Table 1:** **Computation of** ***μ*** ***c*** ***y*** ***c*** ***l*** ***e*** **length for each schedule**

	Schedule A	Schedule B
*C*_41_→*C*_31_	10	2
*C*_31_→*C*_21_	9	1
*C*_21_→*C*_11_	9	1
*C*_11_→*C*_01_	9	1
*μ* *c* *y* *c* *l* *e*	37	5

**Figure 7:**
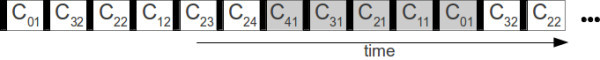
**Resulting schedule B.**

If we use for instance a re-ordering technique (DCR), this will result in the schedule B depicted in Figure 7, favorable to stream *S*_3_, showing a full transaction from source to destination in one TDCS cycle.

Its *macrocycle* is the size of the schedule, which is of 10 base units. *T*_*i*_ is computed according to the methodology presented in Step 5 and is equal to 3. Hence, for *n*=1, considering Schedule A, *t*_*A*_=37+0+0=37. For schedule B, with a DCR, *t*_*B*_=5+0+3=8. The *macrocycle* is equal to 10 for both cases. With our technique it is possible to compute the delay, assuming a collision free environment and maximum theoretical bitrate, an obvious simplification which will always output the shortest time it takes for a flow of data to reach the destination. This method, however, suffices to compute if a re-scheduling is better or not. The root node will then compute all the offsets that result from the new cluster schedule that will serve that stream and reply to the request.

**Figure 8:**
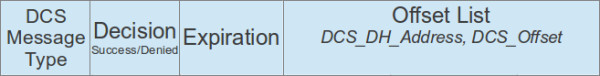
**DCS Reply message format.**

STEP 4 - Reschedule Response; After the computation of the new offsets (time offset between the beginning of the active portions of the parent and child CHs), according to the new schedule, a response is sent in the payload of the periodic synchronization frame. By using the synchronization frame to deliver this information we make sure that all CHs receive the information in a bounded amount of time, since they are not susceptible to contention. The first part (Figure 8) specifies the message type and the response, (request accepted or request denied). The next portion of the frame contains the expiration for that schedule, which is the amount of TDCS cycles the schedule will remain active before returning to the original network schedule. The next portion contains a list with the new offsets expressed in a relative offset concerning the original one and the cluster-head addresses to which these are to be applied.

Only the CHs which received a new offset are part of the content of the response frame. If the node which requested the rescheduling does not find its address among the ones in the response, or if no response is received for more than *DCS_maxWait* cycles, it should hold the data and retry later up to a maximum of *DCS_maxRescheduleRetry* times. The size of *DCS_CH_Address* is implementation specific as well as the *DCS_Offset*, since these variables depend of the protocol.

STEP 5 - Propagation; Each cluster-head, upon reception of the Reschedule Response payload, retrieves its newly assigned offset to their parent and propagates the remaining offset information along the network by placing it in their own synchronization frames, thus propagating the information downstream. The new offset information is then used by the CHs to compute the time for the next synchronization frame. At the next depth, the child router of that cluster-head must wait for the next synchronization frame (with the new offset) from the parent, and synchronize to it. This propagation procedure however can introduce a period over which the network is not fully accessible, with the exception of the branches that remained independent of the CHs which were rescheduled. This holds true for the Cluster Re-ordering technique only (DCR). This is because each CH must wait for the synchronization frame of their parent so that they can align with it and also synchronize their cluster, propagate information and become active, since the offsets are always relative to the parents. However, this delay is bounded and can be easily computed as a function of the TDCS schedule cycles as follows:
4

The inaccessibility time is equal to the depth of the deepest rescheduled CH (*d*_*Ar*_) in the tree minus one, multiplied by the respective duration of one TDCS cycle. This is the amount of time the scheduled branches of the network should be inaccessible. If instead of a DCR technique we use a Bandwidth Redistribution technique, this inaccessibility time is zero. Because the hierarchical order of the schedule is kept, the routers will always receive the synchronization frame of their parent immediately before (assuming a schedule favoring downstream transmission), and within the same TDCS cycle. A failure at the reception of the synchronization frame must place the cluster and its respective children in an idle state to avoid inter-cluster collision. Upon the correct reception of the following synchronization frame the cluster shall resume.

**Figure 9:**
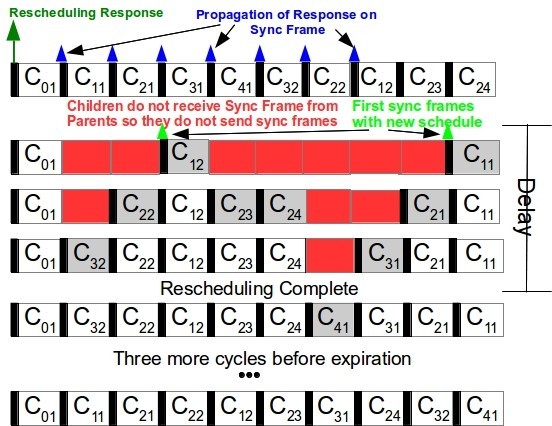
**Example of the DCS.**

STEP 6 - Returning to original schedule; The schedule’s change is not permanent, and the network must roll back to its initial schedule after a defined period of time which we define as the Schedule’s Expiration Period. Because of the inaccessibility period in the DCR technique, each depth will be assigned with a different Expiration so that all depths can change the schedule back to the original in the same cycle. For this reason, Expiration in Step 5 is computed as *Expiration*=*ED*+*Ti*+1, where *ED* is the schedule’s expiration deadline that is application defined (*DCS_Exp_Deadline*) and can be computed from the amount of data to be received, Ti the inaccessibility time. Each CH will later compute its own Expiration by subtracting their depth in the tree. By following this rule, every CH can easily compute when the current schedule expires, just by counting the number of TDCS cycles since their first synchronization frame after the reschedule. For the case of a DBR technique, expiration will be always equal to the *ED*, since the inaccessibility time remains equal to zero. The CHs should activate a counter at the first synchronization frame sent with the new schedule. From this point on, each CH keeps track of the current number of synchronization frames sent by it. When this number is equal to the assigned Expiration value the CH automatically sets its offset to the original and waits for a synchronization frame from its parent to return to the original schedule. Figure 9 describes how this process should work for the example of *S*_3_, after a successful reschedule response. The delay of three cycles due to inaccessibility is depicted as well as the schedule expiration. The first TDCS cycle transmits the new offsets within the DCS Response. Each router resets their internal clock references and waits for a synchronization frame from their parent. *C*_12_ and *C*_11_ are the first to receive this and they transmit their synchronization frames with the new schedule, followed by their child, (*C*_22_,*C*_23_,*C*_24_,*C*_21_).

Next, the CHs at depth three do the same until the last CH at depth four (*C*_41_) is also rescheduled. The schedule is kept for three more TDCS cycles and it expires. All the offsets return to the original schedule in only one TDCS cycle. As observed, the network inaccessibility time is bounded and return to the original schedule is done without much complexity.

Considering energy-efficiency, only the node which makes the DCS Request, and eventually the CHs routing that message, spend an extra quantity of energy, which is equivalent to the transmission of one short data frame, eventually retransmitted in the case of a failure. The setup of the network with the new offsets uses the payload of the synchronization frames that must be transmitted independently of DCS. Thus, it is clear that communications generated by our mechanism, will never lead to energy depletion among the nodes.

## Instantiating DCS in IEEE 802.15.4/ZigBee

### IEEE 802.15.4/ZigBee overview

IEEE 802.15.4 and ZigBee 2005, particularly the synchronized cluster-tree network model, emerge as potential solutions for industrial WSNs, since they enable to fulfill QoS requirements such as energy-efficiency (dynamically adjustable duty-cycle in a per-cluster basis) and timeliness (best effort/guaranteed traffic differentiation and deterministic tree-routing). The IEEE 802.15.4 MAC protocol supports two operational modes that may be selected by the ZigBee Coordinator (ZC), which identifies and manages the whole WSN: i) the non beacon-enabled mode, in which the MAC is simply ruled by nonslotted carrier sense multiple access with collision avoidance (CSMA/CA); and ii) the beacon-enabled mode, in which beacons are periodically sent by the ZC for synchronization and network management purposes. In the beacon-enabled mode, the ZC defines a superframe structure, which is constructed based on the Beacon Interval, which defines the time between two consecutive beacon frames, and on the Superframe Duration (SD), which defines the active portion in the BI, and is divided into 16 equally-sized time slots, during which frame transmissions are allowed. Optionally, an inactive period is defined if BI > SD. During the inactive period (if it exists), all nodes may enter in asleep mode (to save energy). BI and SD are determined by two parameters, the Beacon Order (BO) and the Superframe Order (SO), respectively, as follows:


where aBaseSuperframeDuration = 15.36 ms, (assuming 250 kb/s in the 2.4 GHz frequency band) denotes the minimum superframe duration, corresponding to SO = 0. During the SD, nodes compete for medium access using slotted CSMA/CA in the CAP. For time-sensitive applications, IEEE 802.15.4 enables the definition of a contention-free period (CFP) within the SD, by the allocation of guaranteed time slots (GTSs). Low duty-cycles are achieved by setting small values of the superframe order (SO) as compared to the beacon order (BO), leading to longer sleeping (inactive) periods.

ZigBee defines network and application layer services on top of the IEEE 802.15.4 protocol. In the cluster-tree model, all nodes are organized in a parent-child relationship, network synchronization is achieved through a distributed beacon transmission mechanism and a deterministic tree routing mechanism is used. A ZigBee network is composed of three device types: (i) the ZigBee Coordinator (ZC), which identifies the network and provides synchronization services through the transmission of beacon frames containing the identification of the PAN and other relevant information; ii) the ZigBee Router (ZR), which has the same functionalities as the ZC with the exception that it does not create its own PAN-a ZR must be associated to the ZC or to another ZR, providing local synchronization to its cluster (child) nodes via beacon frame transmissions; and (iii) the ZigBee End-Device (ZED), which neither has coordination nor routing functionalities and is associated to the ZC or to a ZR.

### Integrating DCS in a ZigBee network

**Figure 10:**
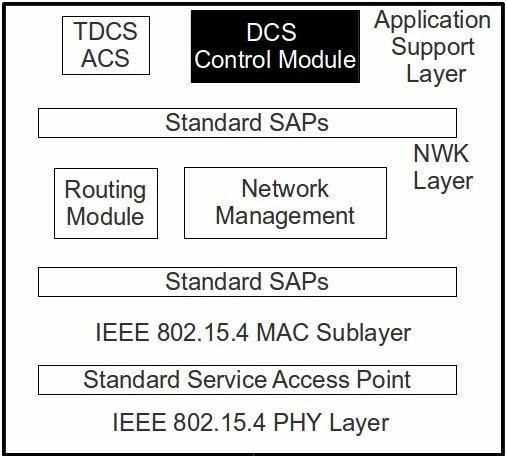
**DCS Architecture.**

The PAN-Coordinator is responsible for receiving the new schedule request from the other cluster-heads and computing the new schedule as described before. A new module was devised to be integrated above the network layer of ZigBee (Figure 10), at the Application Support Layer. This new module, DCS, is responsible for managing the DCS mechanism, in regards to the beacon payload creation (for propagating offset information), computing and changing the offset information for the lower layers, and computing the schedules and corresponding expiration.

At network setup time, the TDCS algorithm is applied to the tree, setting up the base schedule. As the nodes gather data, they can direct streaming requests at the PAN Coordinator. The PAN-Coordinator will evaluate these requests according to what is described in Step 3 of Section “The DCS communication protocol”. If the result is positive, it will compute the new schedule and setup the Rescheduling Response to be placed in the IEEE 802.15.4 Beacon Payload. The next Beacon frame will carry this information. As Beacons are transmitted between the several clusters, the new schedule information is propagated among the tree and all the nodes will know a DCS Rescheduling is occurring just by parsing the received Beacon. This is important since in the next BI, many nodes will fail to receive a Beacon from their parent, due to the inaccessibility time described in Step 4 of the DCS Communication Protocol. This will be specially visible in the deepest nodes of the rescheduled branch. If no information concerning the status of the process was propagated, the nodes could assume they had lost their parent, receiving a SYNC-LOSS.indication from the respective MAC layer, and would try an Association procedure to another potential parent. By knowing this in advance, they can disable this process for (*Depth*−1)∗*BI* amount of time, which is the maximum time the rescheduling should take per Depth, after which, the Device will re-enable the re-association procedure after the SYNC-LOSS.

Upon reception of their parent’s Beacon, the ZigBee Cluster-Heads, will search for their address among the Rescheduling information at the Beacon Payload to learn the new offset. Then, they will trigger the DCS Module generating a *DCS-NEW-SCHEDULE.indication*, and set their own Beacon Payload with the remaining information of the Rescheduling Response to propagate the information to the children down the tree. Having done this, the DCS Module, will issue a SYNC.request to the Network Layer to resynchronize with the corresponding parent, and after a synchronization an *MLME-START.request*.

The *MLME-START.request* primitive, depends of the rescheduling technique to be used. If a Re-ordering technique is to be used, then the CH will used a *DCS-RESTART-ROUTER.request*, with the new offset information. This new interface is similar the standard *NLME-RESTART-ROUTER.request*, except no change is done to the other parameters of the stack. The objective is to simply turn the routing functionality on. If a Bandwidth reallocation is to be done, then the request will also change the Superframe Order parameter of the stack to reflect the bandwidth change. The system timers at the MAC layer, upon reception of this request are automatically updated with the new Superframe Order. Upon the reception of a Beacon from the parent, the ZigBee Router will automatically resynchronize and resume its work.

When the DCS Module is triggered, the Schedule Expiration is also computed according to what is described in Step 6 of Section “The DCS communication protocol”, and a counter (*DCS-Expiration-timer*) is triggered with that value. When this counter expires, the DCS Module automatically repeats the *DCS-RESTART* process with the old offset values, returning to the initial values. These are stored in a database, *DCS-Initdb*, which contains the initial offset and Superframe Order values. As described, the implementation of the DCS mechanism does not involve major changes to the protocol. In fact, only a couple of new interfaces are to be added to the ZigBee NWK implementation to enable the DCS functionalities. One is triggered upon reception of a new schedule (*DCS-RESTART-ROUTER.request*) after the regular parsing of a beacon frame, and another (*NLME-RESTART-ROUTER.request*) which is a replication of the standard *NLME-START-ROUTER.request*. All of the DCS mechanism implementation is taken as an independent module to the Application Support Layer to avoid imposing substantial changes to the NWK layer.

## Performance evaluation

### Application scenario

Structural Health Monitoring (SHM) and damage identification at the earliest possible stage have been receiving increasing attention from the scientific community and public authorities (Superstructures 2010). Service loads, accidental actions and material deterioration may cause damage to the structural systems, resulting in high administrative costs for governments and private owners and, in some situations, loss of human lives. As such, there is an enormous eagerness to add sensing/actuating capabilities to physical infrastructures like bridges, tunnels and buildings, turning them into “smart structures” able to detect and respond to abnormal situations. However, there is still a lack of ready-to-use and off-the-shelf WSN technologies able to fulfil the most demanding requirements of SHM applications, such as stringent time synchronization of all sensors’ measurements, highly reliable timely measurements and data communications. In this line, we designed a prototype system for SHM ((Severino et al. 2010) and (Aguilar et al. 2011)), capable of coping with these SHM requirements while supporting network scalability.

This application presents interesting dynamics that could be improved by the use of the DCS mechanism. Besides its requirement of tight node synchronization and low latency downstream control, the application generates a large amount of sensing data that must be handled by the network in an upstream direction. These two modes of operation can be supported and see their performance improved by the use of the DCS mechanism by minimizing both end-to-end delays and overall transmission time.

**Figure 11:**
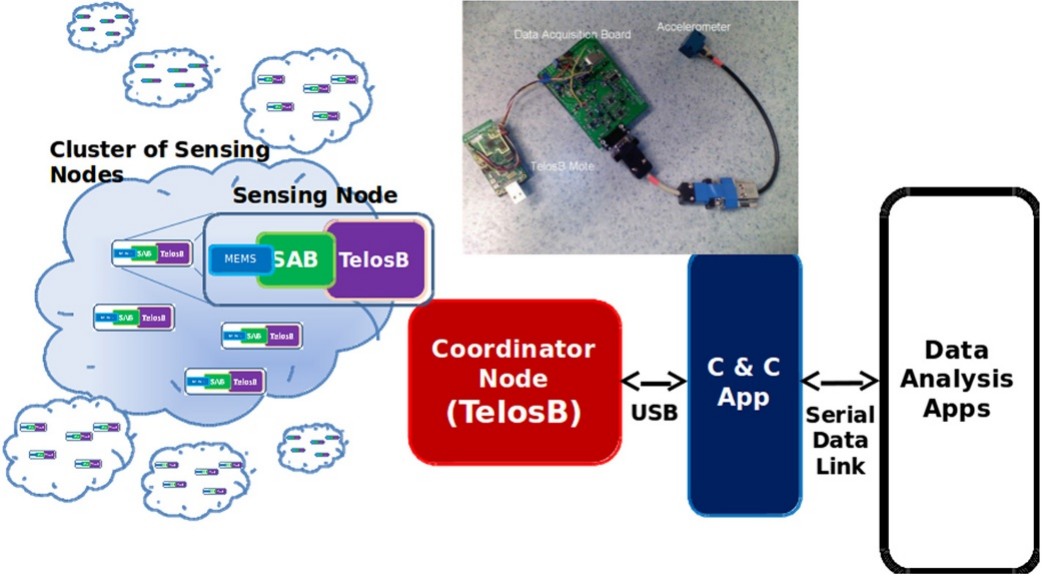
**SHM System architecture.**

Its system architecture was designed to sample in a synchronized fashion multiple accelerometers placed at different locations in a physical structure and forward this data to a central station (PAN-Coordinator) for later processing using a IEEE 802.15.4/ZigBee Cluster-Tree network topology. Each Sensing Node is composed by a TelosB node (MEMSIC 2014) with a signal acquisition board, with a 24 bits DAC, attached to a MEMS 3-axis acceleration sensor (Figure 11).

The Coordinator Node (Figure 11) supervises the network and nodes’ activities and guarantees a tight synchronization between all nodes. It also forwards the configuration parameters and dispatches the acquired data to the Command & Configuration Application (C&C App), which provides the system user with a human-machine interface (HMI) to configure the system and also an application programming interface (API) to integrate the WSN system with the data processing/analysis applications. When the data acquisition finishes, the Sensing Nodes are pooled in turn for the sensing data. Depending on the sampling rate, a large volume of data is transmitted to the Coordinator Node, which will forward it to a PC for processing. With reduced latency, it could be possible to carry out data processing simultaneously. Nevertheless, engineers can rely on data post processing, as long as data arrives within the minimum possible period, since they need several acquisition runs to be carried out.

The network is setup according to Figure 1 network topology and the Sensing Nodes are spread into different clusters. In Figure 1, the addresses next to the nodes represent the Cluster-Heads’ ZigBee NWK addresses. The initial schedule favors downstream communications. This is made so that the PAN-Coordinator, after setting up all the nodes in the network, is able to start and stop the data acquisition on all the nodes simultaneously, in one TDCS cycle. This is mandatory for the application so that the results are coherent. If the initial schedule was kept to poll the date from the sensors, the assessment could last minutes up to several hours, depending on the cluster the stream originated from.

We aim at changing the network’s TDCS schedule to improve on this behaviour, by (1) reducing end-to-end latency, eventually allowing for simultaneously data analysis, using the DCR technique and (2) by accelerating the data transfer from the Sensing Nodes to the PAN-Coordinator using DBR technique.

### Experimental setup

The DCS mechanism was evaluated through simulation and experimentally using our SHM system. For carrying out the simulation analysis, the DCS mechanism was implemented over the Open-ZB Zigbee Model (ZigBee-Alliance 2005), and simulated with the OPNET Modeler simulation software. A network topology like the one shown in Figure 1 with nwkMaxChildren (Cm) = 3, nwkMaxDepth (Dm) = 5, and nwkMaxRouters (Rm) = 2, was setup and the application layer of the node was set to generate traffic at a rate correspondent to a sampling rate of 100 Hz which is recommended for fine-grained structural health monitoring (Severino et al. 2010). For maintaining uniformity along this paper, in the analysis we always consider stream *S*_3_, which originates at router *C*_41_ in Figure 1 and constitutes the worst-case for the initial TCDS schedule. Several analysis to evaluate the performance of the two techniques were carried out, with a special attention to two metrics: end-to-end delays and overall stream transmit duration.

To carry out the experimental validation over the SHM application, the DCS module was implemented in TinyOS over the Open-ZB IEEE 802.15.4/ZigBee stack (Cunha et al. 2007). A ZigBee network with 12 TelosB motes was setup in a configuration replicating the one depicted in Figure 1, using BO=8 and SO=4, with one PAN-Coordinator connected to a PC through a USB connection, and nine Routers each forming their own cluster. Two Sensing Nodes (End Devices) were associated to the Router at Depth 4 (address 0x0004) to generate sensing data for later retrieval. To reduce costs, the Sensing Nodes were used without the accelerometer modules. Instead, timers at the application layer were used to emulate real SHM traffic at different sampling rates. Both DCS techniques were implemented and tested to validate our work, although the most important technique for this specific SHM application is the DBR, which as shown before can greatly reduce the overall stream transmission time. A base scenario, without any schedule improvement, was also setup to measure the improvement.

### Performance results

#### End-to-end Delay Analysis

To understand the impact of the first technique we did one hundred simulation runs, 10 minutes each, of the network with different BO settings (from BO = 8 up to BO = 12), simulating a larger network, with the initial scheduling and using the cluster re-ordering technique. The maximum end-to-end delays were measured for packets transmitted from a Sensing Node associated to Router 0x0004, at Depth 4, (*S*_3_ in Figure 1), to the PAN-Coordinator, with no extra traffic on the network. This transmission is the worst-case for the initial TDCS schedule. In the simulation platform, frame size was set to 800 bits, and Packet Inter-arrival Time was set to 0,06 seconds to emulate the arrival of Sensing Data at the Sensing Node’s serial port (this was verified experimentally).

**Figure 12:**
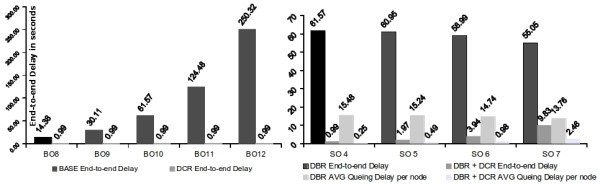
**Stream end-to-end delays - simulation.**

Figure 12, shows the simulation maximum end-to-end delay results for the different BO using the DCR technique. Superframe order is fixed to SO = 4 for the case of the results in the left. Notice the decrease on the delay achieved by simply re-ordering the schedule. We can achieve a reduction in the end-to-end delays in the order of 13 seconds for BO = 8 and even several minutes as the BO increases with the size of the network, reaching 4 minutes for the case of BO = 12, to approximately one second. The end-to-end delays with DCR remain constant despite the different BO settings. This is expected since although the network increases, the transmission of a packet is completed in only one BO cycle. Since the Bandwidth of the routers is also the same, the end-to-end delay should remain constant and thus independent of the network size.

To understand the impact of the Bandwidth Reallocation technique (DBR), on the end-to-end delay, the initial schedule’s ordering was maintained, and BO increased to 10. As the available bandwidth of the Superframe increased, it was distributed using DBR among the Routers involved on the stream, changing from SO=4 up to SO=7. Figure 12 on the right, presents the results for *S*_3_ in Figure 1 concerning end-to-end delay.

There is a slight but not significant decrease of the end-to-end delay as the SO are increased. Since the Routers increase their SO, the unused part of the Superframe was reduced and thus there is a better use of the Superframe bandwidth. This reduces the time the packet must remain in the queue at each router, waiting for the next Superframe to be transmitted to the parent, thus slightly reducing the overall end-to-end delay. This is visible in the figure in the right showing how the average queuing delay decreases as the SO increases. In comparison, the DCR technique presents a much higher impact on the end-to-end delay as expected, decreasing for the case of BO/SO = 10/5, the delay from 60,95 to 1,97 seconds, a decrease of 96,7%. In fact, for its worst case of BO/SO = 10/7, it still represents a decrease of 82,14% concerning the DBR technique.

There is however, a slight increase in the end-to-end delay when using the DBR+DCR techniques as the SO increases. Although, there is a re-ordering of the schedule according favouring upstream traffic, and a redistribution of the unused bandwidth, the increase in SO implies a larger time a packet must wait in queue at each router, waiting to be transmitted to the parent, in comparison to the cases with lower SO. Using the DBR technique is thus not recommended when one wishes to significantly reduce the end-to-end delay in the application.

**Figure 13:**
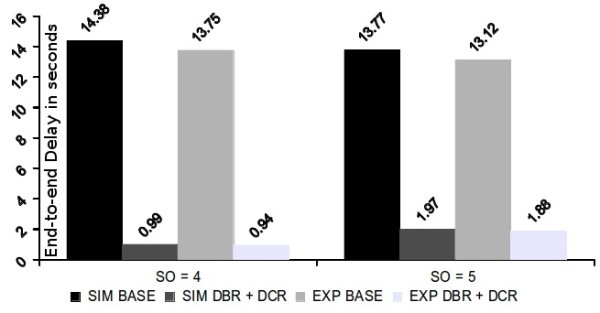
**Stream end-to-end delays - experimental and simulation.**

Figure 13 shows the comparison between simulation and experimental results. As observed, the behaviour previously observed in simulation is replicated in the experimental evaluation with minor differences. However for the base schedule, experimental delay was slightly different. This has to do with the different duration of the Beacon Order on the experimental platforms, which is of 3,75 seconds instead of the theoretical 3,932 seconds, due to the lower timer granularity.

**Figure 14:**
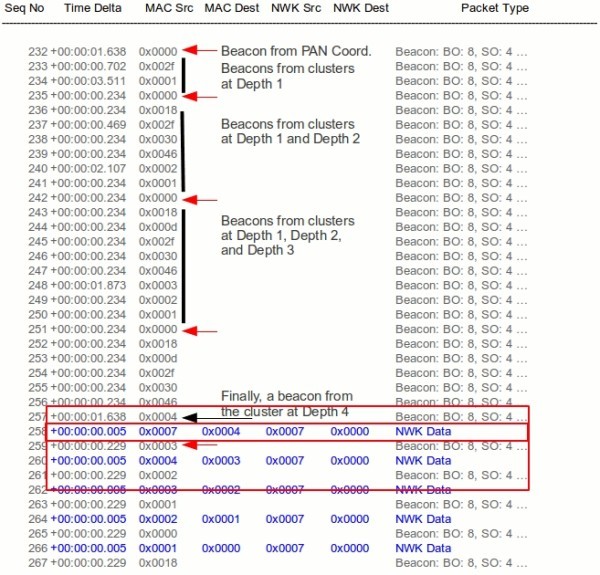
**Output from the packet analyzer showing the DCR technique.**

A Daintree Networks 2400E Sensor Network Analyzer (Daintree Networks 2014) was used to log all the communications during the experimental evaluation. Figure 14, shows its log after a successful reschedule response. Part of the output related to the network setup and DCS communication was omitted to save space, but can be seen in (Severino et al. 2013b). The beacons from the PAN Coordinator are signaled with a red arrow. When all the Routers receive their new offset information in the DCS Reschedule Response message, they immediately stop sending beacons and wait for their parent’s beacon to synchronize to it. The first beacon comes from the PAN Coordinator which maintains its period. Next, Routers at Depth one are the firsts to synchronize to it using the new offsets. Notice the Packet Analyzer time stamp, showing the new relative offsets. Now that the Depth one Routers transmitted their beacons, the next level ones (Depth two) can also synchronize. The process continues until the all the Routers are synchronized. At this point, the Sensing Nodes (0x0007 in the example) start transmitting data which will be forwarded until it reaches the sink.

#### Stream overall transmission time

Like previously mentioned, minimizing the overall transmission time is quite important, in this SHM application, where large amounts of data must be transmitted in the less amount of time possible. To analyze this metric, in the simulation platform we generated scenarios with different volumes of sensing data, corresponding to short 10 and 30 seconds runs and runs with 1, 5, 10 and 30 minutes, in the SHM system. Those values were also tested in the experimental platform. During this time there was no more traffic in the network, so that collisions were not possible, not to interfere with the experiment.

**Figure 15:**
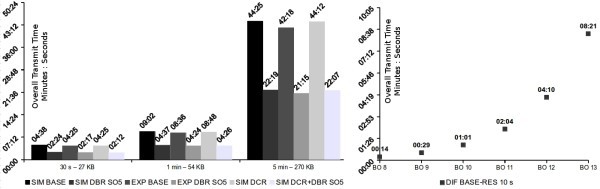
**Stream transmit duration.**

Figure 15 shows the simulation and experimental results of the transmission time for sampling durations of 30, 60 seconds and 5 minutes using the DCR and DBR techniques for SO=4 and SO=5 in regard to the base schedule. As shown, the DBR technique presents the best result in decreasing the overall transmission time, representing a decrease close to 50%, as expected when the available bandwidth is doubled on the Routers, to SO = 5. For the case of 1 minute of sampling time, using the DBR technique alone reduced the overall transmission time from nine minutes to 4 and a half minutes, a decrease of 49%.

Interestingly, the DCR technique also decreases the overall transmission time, but not in a significant way. It decreases it about 14 seconds for this particular case of BO=8, and it is constant for every SO setting, independently of the amount of data to be transmitted. This small difference, however should not be neglected. For larger BO, the impact of this increases as shown in Figure 15 in the right, reaching 8 minutes for BO=13. This happens because of the impact of the reduced end-to-end delay at the beginning of the transmission, due to the re-ordering of the clusters’ schedule. Because of this, the transmission will end sooner. As the BO increases, the impact of this is higher since the duration of the TDCS cycle also increases.

Experimentally, concerning the DBR technique, results show a reduction on the overall transmission time in the order of 49%, again quite close to simulation results, while further reductions can be achieved by increasing the SO per cluster. Concerning the network inaccessibility time, as predicted, it was bounded to three TDCS cycles, which is the time it takes for the whole network to resynchronize with the new schedule. This can be confirmed in the Packet Analyzer output files available in (Severino et al. 2013b).

## Conclusions

Changing the resource allocation of a Cluster-based WSN on-the-fly, without imposing long inaccessibility times, represented a major challenge, hindering the deployment of many WSN applications. In this paper we presented a solution to this problem, enabling networks to self-adapt to changing traffic flows, improving the QoS by redistributing the available bandwidth and minimizing latency, assuming a given schedule based on a time-division strategy.

We presented two techniques achieving a reduction of the end-to-end delay from a leaf node to the sink of 93%, and a decrease of the overall data transmit period of 50% although a higher impact can be achieved with other network settings. Importantly, our methodology was applied to a real-world WSN-based Structural Health Monitoring system, showing that it can be easily implemented under the IEEE802.15.4/ZigBee set of protocols with minor add-ons and can run in general purpose WSN platforms such as the TelosB motes.

In the near future, we aim at deploying the SHM system fitted with DCS in the field, supporting civil engineers that must carry out professional structural health monitoring work. We are also aiming at providing support for conflicting traffic flows in the network.
